# Azanitrile Cathepsin K Inhibitors: Effects on Cell Toxicity, Osteoblast-Induced Mineralization and Osteoclast-Mediated Bone Resorption

**DOI:** 10.1371/journal.pone.0132513

**Published:** 2015-07-13

**Authors:** Zhong-Yuan Ren, Irma Machuca-Gayet, Chantal Domenget, Rene Buchet, Yuqing Wu, Pierre Jurdic, Saida Mebarek

**Affiliations:** 1 State Key Laboratory of Supramolecular Structure and Materials, Jilin University, Changchun, 130012, China; 2 Université de Lyon, Villeurbanne F-69622, France; 3 Université Lyon 1, Villeurbanne F-69622, France; 4 CPE Lyon, Villeurbanne F-69622, France; 5 INSA-Lyon, Villeurbanne F-69622, France; 6 Institut de Chimie et de Biochimie Moléculaires et Supramoléculaires, Villeurbanne F-69622, France; 7 CNRS UMR 5246, Villeurbanne F-69622, France; 8 Ecole Normale Supérieure de Lyon, Lyon F-69634 France; 9 Institut de Génomique Fonctionnelle de Lyon, Lyon F-69634 France; 10 CNRS UMR3444, Lyon F-69634 France; Inserm U606 and University Paris Diderot, FRANCE

## Abstract

**Aim:**

The cysteine protease cathepsin K (CatK), abundantly expressed in osteoclasts, is responsible for the degradation of bone matrix proteins, including collagen type 1. Thus, CatK is an attractive target for new anti-resorptive osteoporosis therapies, but the wider effects of CatK inhibitors on bone cells also need to be evaluated to assess their effects on bone. Therefore, we selected, among a series of synthetized isothiosemicarbazides, two molecules which are highly selective CatK inhibitors (CKIs) to test their effects on osteoblasts and osteoclasts.

**Research Design and Methods:**

Cell viability upon treatment of CKIs were was assayed on human osteoblast-like Saos-2, mouse monocyte cell line RAW 264.7 and mature mouse osteoclasts differentiated from bone marrow. Osteoblast-induced mineralization in Saos-2 cells and in mouse primary osteoblasts from calvaria, with or without CKIs,; were was monitored by Alizarin Red staining and alkaline phosphatase activity, while osteoclast-induced bone resorption was performed on bovine slices.

**Results:**

Treatments with two CKIs, **CKI-8** and **CKI-13** in human osteoblast-like Saos-2, murine RAW 264.7 macrophages stimulated with RANKL and mouse osteoclasts differentiated from bone marrow stimulated with RANKL and MCSF were found not to be toxic at doses of up to 100 nM. As probed by Alizarin Red staining, **CKI-8** did not inhibit osteoblast-induced mineralization in mouse primary osteoblasts as well as in osteoblast-like Saos-2 cells. However, **CKI-13** led to a reduction in mineralization of around 40% at 10–100 nM concentrations in osteoblast-like Saos-2 cells while it did not in primary cells. After a 48-hour incubation, both **CKI-8** and **CKI-13** decreased bone resorption on bovine bone slices. **CKI-13** was more efficient than the commercial inhibitor **E-64** in inhibiting bone resorption induced by osteoclasts on bovine bone slices. Both **CKI-8** and **CKI-13** created smaller bone resorption pits on bovine bone slices, suggesting that the mobility of osteoclasts was slowed down by the addition of **CKI-8** and **CKI-13**.

**Conclusion:**

**CKI-8** and **CKI-13** screened here show promise as antiresorptive osteoporosis therapeutics but some off target effects on osteoblasts were found with **CKI-13**.

## Introduction

**Fig 1:**
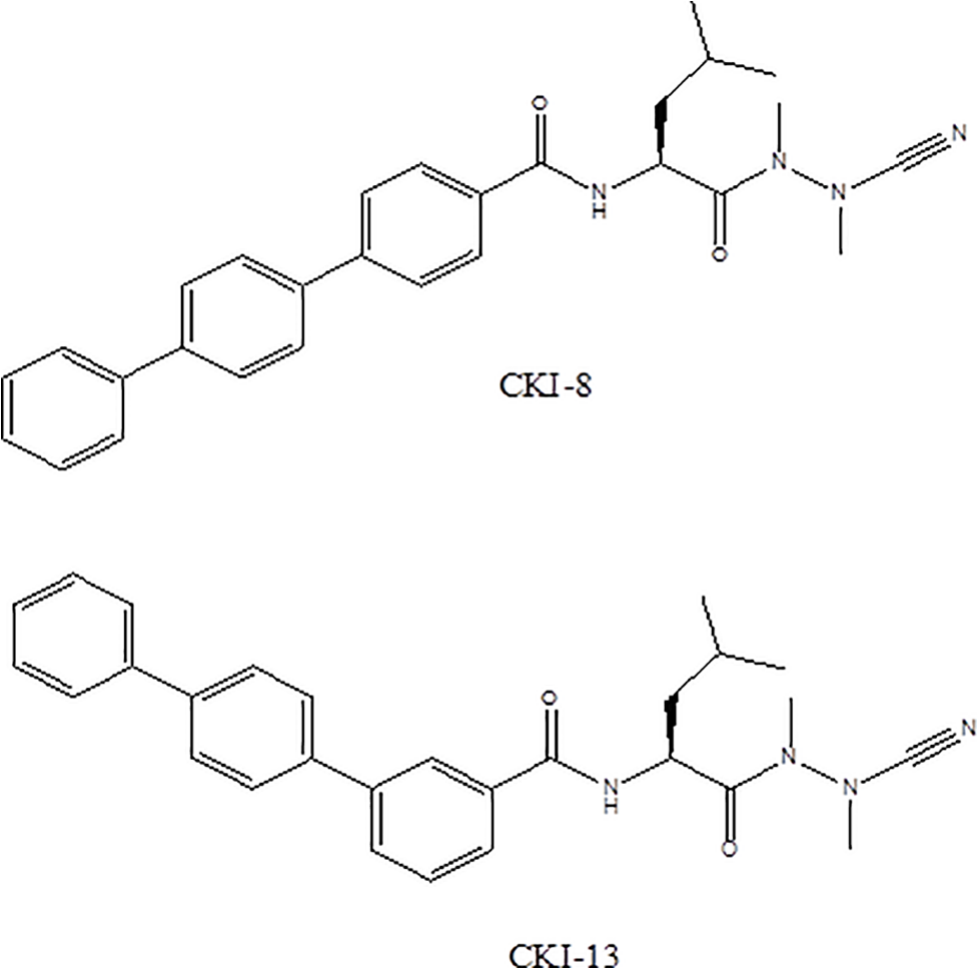
Structures of CKI-8 and CKI-13.

Osteoporosis is a common medical and socioeconomic threat characterized by a systemic loss of bone mass, strength, and microarchitecture, which increases the risk of fragility fractures [1, 2]. Detailed knowledge of bone biology [3] with molecular insights into the communication between bone-forming osteoblasts and bone-resorbing osteoclasts, as well as the signaling networks involved, has led to the identification of several therapeutic targets. Among these, drug treatment strategies have been developed, aimed at inhibiting excessive bone resorption and at increasing bone formation. With the exception of parathyroid hormone and its analogs, all agents currently used in the treatment of osteoporosis, such as bisphosphonates, selective estrogen receptor modulators, and the anti-RANKL antibody, act primarily by decreasing osteoclast-mediated bone resorption [4]. One of the most promising drug treatments is based on the specific inhibition of the osteoclast protease cathepsin K (CatK) to slow down bone resorption [5]. CatK is a collagenase and the predominant papain-like cysteine protease expressed in osteoclasts, [5, 6]. The inhibition of bone resorption observed in human and animal models deficient for CatK indicated that this enzyme is a suitable target for intervention by small molecules that might be used as therapeutic agents in osteoporosis. Targeted disruption of the CatK gene in mice produced a high bone mass phenotype [7, 8] while overexpression of CatK increased bone turnover and decreased trabecular bone volume [9]. Therefore, considerable effort has been put into developing highly selective and orally applicable CatK inhibitors (CKI) [10]. Four CKIs, Relacatib, Balicatib, MIV-711 and Odanacatib (ODN) have been evaluated as possible drug therapies to prevent bone resorption [11–13]. Relacatib was discontinued following a Phase I evaluation that showed possible drug–drug interactions with the commonly prescribed medications paracetamol, ibuprofen, and atorvastatin [14]. Balicatib trials were discontinued due to dermatologic adverse effects, including a morphea-like syndrome [14]. MIV-711 has been evaluated successfully in a Phase I of clinical research for the treatment of osteoarthritis and other bone related disorders [13]. Only ODN has presently reached phase III of clinical research [14–17]. In preclinical research, ovariectomized monkeys and rabbits treated with ODN showed substantial inhibition of bone resorption markers along with increases in bone mineral density (BMD). Phase I and II trials conducted in postmenopausal women showed that ODN to be safe and well tolerated [14]. Although developed as antiresorptive agents, several compounds show lysosomotropic effects [16], cutaneous adverse effects and anabolic activity [18], which are intrinsically related to the selectivity of inhibitors toward CatK. Therefore, alternative compounds having better selectivity toward CatK may complement the use of CKIs in bone resorption therapy. Typically, CKIs are mainly derived from peptides or peptidomimetic structures, which generally contain electrophilic entities prone to covalently interact with the cysteine-thiol moiety in enzymes. With the rapid development of powerful and selective inhibitors for CatK, azapeptide nitriles have attracted much attention due to their extremely potent inhibition albeit with a relatively low selectivity [19–23]. Among these, proteolytically stable azadipeptide nitriles have been developed, with picomolar Ki value towards the therapeutically relevant cathepsins B, K, L and S with which they form reversible isothiosemicarbazide adducts [24–26]. Recently, we synthesized two series of candidate azanitrile inhibitors that were selected for their inhibition against human CatK activity in vitro [24]. One inhibitor (**CKI-13**) from the first group, resulted in a picomolar Ki values with remarkable selectivity over the cathepsins B and S. The other inhibitor (**CKI-8**) from the second group, led to further improvement in the CatK selectivity by shortening the length of P3-P2 linker (Fig 1).

These azanitriles inhibit cysteine proteases by forming a reversible isothiosemicarbazide adduct resulting from the nucleophilic attack of a thiol on the carbon-nitrogen triple bond. In order to provide a proof of concept of the potential use of our CKIs [24] in drug treatments for osteoporosis, as well as to consolidate the efficiency/biological evaluation of these compounds, prior to preclinical trials, we compared the effects of two inhibitors, CKI-8 and CKI-13 with those of the commercial inhibitor E64 on cell viability, osteoblast-induced mineralization and osteoclast-induced bone resorption. Mineralization tests on osteoblasts consisting in Alizarin-Red staining to detect calcium nodules and in alkaline phosphatase activity, a biomarker of mineralization, served to verify the off-target effects.

## Materials and Methods

### Ethics statements

All experiments were carried out according to the guidelines laid down by the French Ministère de l’Agriculture (n° 87–848) and the E.U. Council Directive for the Care and Use of Laboratory Animals of November 24th, 1986 (86/609/EEC). Animal experiments were performed under the authorization n°69-266-0501 (INSA-Lyon, DDPP-SV, Direction Départementale de la Protection des Populations—Services Vétérinaires du Rhône), according to the guidelines laid down by the French Ministère de l’Agriculture (n° 87–848) and the E.U. Council Directive for the Care and Use of Laboratory Animals of November 24th, 1986 (86/609/EEC). MLC (n°692661241), AG (n°69266332) and COS (n°69266257) hold special licenses to experiment on living vertebrates issued by the French Ministry of Agriculture and Veterinary Service Department. The experiments were realized on euthanized animals by dislocation of cervical vertebra, which did not require surgery and were not painful.

### Primary osteoblast cells

Primary osteoblast cells were enzymatically isolated from calvaria of 5–6 day old mice (C57BL/6J strain). Cells were isolated using sequential digestion at 37°C with 0.05% trypsin/ ethylenediaminetetraacetic acid (EDTA) for 20 min and then with 0.8 U mL^-1^ liberase for 20 min. The first two digestions were discarded, and the cells obtained after two digestions (each time incubated with 0.8 U mL^-1^ liberase for 45 min) were collected. The cells were plated at 100,000 cells/well in 12-well plates (Corning Inc) in Dulbecco modified Eagles medium (DMEM) containing 15% (mL:mL) fetal bovine serum (FBS) and 24h later switched to DMEM containing 10% FBS (mL:mL) supplemented with 50 μg mL^-1^ L-ascorbic acid (AA) for six days. They were then transferred to DMEM containing 10% FBS (mL:mL) supplemented with 50 μg mL^-1^ L-ascorbic acid (AA) [27–30] with 0.1% DMSO (mL:mL) and 7.5 mM β-glycerophosphate (β-GP) without (control) or with CKI for one more week. AA and β-GP are two osteogenic factors commonly used to stimulate osteoblast differentiation and mineralization [27–30]. Cultures were maintained in a humidified atmosphere consisting of 95% air and 5% CO_2_ at 37°C.

### Human osteosarcoma Saos-2 cells

Saos-2 cells (ATCC HTB-85) were cultured in so-called growth medium consisting of DMEM (ATCC) supplemented with 10% FBS (mL:mL) (Gibco). For the MTT test (3-(4,5-dimethylthiazol-2-yl)-2,5diphenyltetrazolium bromide), Saos-2 cells were plated in 96-well plates (10,000 cells/well) in growth medium supplemented with 0.1% DMSO (mL:mL), 50 μg mL^-1^ AA and 7.5 mM β-GP for three days without (control) or with CKI. For mineralization and TNAP assays, cells were plated in 12-well culture plates at 100,000 cells/well in growth medium supplemented with 0.1% DMSO (mL:mL), 50 μg mL^-1^ AA and 7.5 mM β-GP for eight days without (control) or with CKI.

### The transformed murine monocytic cell line RAW 264.7

Murine macrophage RAW 264.7 cells, responds to RANKL stimulation *in vitro* to generate bone resorbing multinucleated osteoclast (RAW-OCs) with the hallmark characteristics expected for fully differentiated osteoclasts (OCs) [31–33]. Cell line Raw 264.7 (ATCC, Manassas, VA, USA) was cultured in DMEM supplemented with 10% FBS (mL:mL), so-called growth medium. For MTT tests, cells were plated in a 96-well plate (10,000 cells/well) in growth medium containing 0.1% DMSO (mL:mL), 20ng mL^-1^ recombinant mouse receptor-activator of NF-κB ligand (mRANK-L, PAP; SFR Biosciences), without (control) or with CKI for three days. For zymography, cells were plated in a 6-well plate (350,000 cells/well) in growth medium containing 20 ng mL^-1^ mRANK-L for five days.

### Osteoclasts

Osteoclasts from bone marrow cultures from posterior limbs were collected from 7–9 week-old mice (C57BL/6J strain). Cell suspensions were prepared by flushing bone marrow cells using complete medium (α-MEM) (Invitrogen, Cergy Pontoise, France) supplemented with 10% FBS (mL/mL), and 2 mM L-glutamine (Invitrogen)). Mononuclear cells isolated using lymphocyte separation medium (EuroBio, Courtaboeuf, France) were seeded in complete α-MEM medium supplemented under osteoclastogenic conditions with 30 ng mL^-1^ mRANKL and 25 ng mL^-1^ recombinant mouse macrophage-colony stimulating factor (PAP; SFR Biosciences), in a so-called differentiation medium. After five days, mature osteoclasts were enumerated under a microscope on the basis of the number of nuclei (n ≥3).

### Cell viability assay

The viability of cultured cells (Saos-2 or RAW 264.7 cell lines or osteoclast differentiated from bone marrow cells) was measured using the MTT colorimetric assay (Roche Diagnostics, Meylan, France) as described previously [34]. Then the MTT labeling reagent (0.5 mg mL^-1^ final concentration) was added to each well. The cells were further incubated for 4h. 100 μl of solubilization solution (10% SDS (g:mL) in 0.01M HCL) were then added and plates were allowed to stand overnight at 37°C in a humidified atmosphere. Cell viability was directly related to the difference in absorbance measured at 550 and 690 nm using a Tecan Infinite M200 (Salzburg, Austria) micro-titre plate reader. Results were normalized relative to their respective controls taken as 100. For each inhibitor/cell combination, three distinct sample pools were analyzed in a triplicate manner (n = 9).

### Calcium nodule detection

Cell layers (Saos-2 cells or primary osteoblasts) were washed with phosphate-buffered saline (PBS) (PAA) and stained with 0.5% (g:mL) Alizarin Red-S (AR-S) (Sigma) in PBS (pH 5.0) for 30 min at room temperature. After washing, cell cultures were destained with 3.6% (g:mL) cetylpyridinium chloride (Sigma) in PBS pH 7.0 for 60 min at room temperature. The AR-S concentration was determined by measuring the absorbance at 562 nm [29].

### Tissue-nonspecific alkaline phosphatase activity assay

Cells (Saos-2 cells or primary osteoblasts) were harvested in 0.2% (ml:mL) Nonidet P-40 and disrupted by sonication. The homogenate was centrifuged at 1,500 g for 5 min, and TNAP activity in the supernatant was measured using p-nitrophenyl phosphate *p*NPP as substrate at pH 10.4 and recording the absorbance of nitrophenolate at 405nm (ԑ is equal to 18.8 mM ^-1^ cm ^-1^) [35]. In the same lysates, the protein content was determined using a bicinchoninic acid (BCA) assay (Pierce). Results are shown as μmol para-nitrophenolphosphate min^-1^mg of total protein^-1^.

### Zymography of cathepsin K in cell lysates [36]

A cell extraction buffer (20 nM Tris–HCl pH 7.5, 5 mM ethylene glycol tetraacetic acid, 150 mM NaCl, 20 mM β-glycerol phosphate, 10 mM NaF, 1 mM sodium orthovanadate, 1% (mL:mL) Triton X-100, and 0.1% (mL:mL)Tween 20) was added to the cells (osteoclast differentiated from RAW 264.7 macrophage or from bone marrow cells as indicated). Equal amounts of protein were resolved by 12.5% SDS–polyacrylamide gels containing 0.2% (g:mL) gelatin, a CatK substrate. Gels were washed in 65 mM Tris buffer (pH 7.4) with 20% (mL:mL) glycerol for 10 min. Gels were then incubated for 30 min at room temperature in activity buffer containing 100 mM sodium phosphate buffer pH 6.0, 1 mM EDTA and 2 mM dithiothreitol. Then this activity buffer was exchanged for fresh activity buffer containing 0.1% DMSO (mL:mL) without (control) or with CKI for 24 h incubation at 37°C. The gels were rinsed twice with deionized water and were incubated for 1 h in Coomassie stain (10% mL:mL acetic acid, 25% mL:mL isopropanol, and 4.5% mL:mL Coomassie blue) This was followed by destaining (10% mL:mL isopropanol and 10% mL:mL acetic acid). White spots on the SDS-polyacrylamide gel with gelatin indicated the disappearance of gelatin due to its digestion by CatK revealing CatK activity, while blue coloration indicated gelatin due to the lack of CatK activity. Gels were scanned using a Canon scanner. Densitometry was performed using Image J software (developed by the National Institutes of Health), and the IC_50_ values for CKI-8 and CKI-13 inhibition on human CatK were calculated based on the results of gelatin zymography.

### TRAP staining of differentiated cells from RAW 264.7 cells and from bone marrow cells

To confirm the generation of multinucleated osteoclast cells, the differentiated cells from RAW 264.7 cells and from bone marrow cells were stained for TRAP using the TRAP-staining kit (Sigma-Aldrich), according to the manufacturer’s instructions. TRAP-positive multinucleated (3 or more nuclei) osteoclasts were visualized by light microscopy and photographed. Each osteoclast formation assay was performed independently at least 3 times.

### Resorption pit assay

Assessment of bone resorption per single osteoclast (resorption index) was performed as described [37]. Briefly, osteoclasts differentiated from bone marrow from mice were differentiated by adding 30 ng mL^-1^ mRANKL and MCSF at 25 ng mL^-1^. After 5 days of differentiation, they were detached from plastic plates using PBS 25 mM EDTA and replated on bovine bone slices (50,000 cells/well) in 96-well plates. They were treated with 30 ng mL^-1^ mRANKL and MCSF at 25 ng mL^-1^, in the presence or absence of CKI. After 48h, mature cell numbers were determined by staining cells for TRAP. Osteoclasts were scraped from the slices using cotton buds, and slices were stained with a 1% (g:mL) toluidine solution. Images were acquired using a widefield microscope with side illumination, followed by quantitative analysis of the resorption area using Image J software 1.44p (NIH, USA). The results for each bone slice were expressed as the ratio of the area of resorption to the number of mature osteoclasts, and were normalized relative to their respective controls (without inhibitors), taken as 100 (corresponding to 3914 μm^2^/ osteoclast). For each inhibitor in the cells three distinct sample pools were analyzed in a duplicate manner (n = 6).

### Statistics

A Student t test (t test) was performed using Sigma test software. Results were considered statistically significant at p-values <0.05 indicated by (*) while p<0.01 is indicated by (**) in all figures.

## Results

### Cytotoxicity of CKI-8 and CKI-13 on cells

**Fig 2:**
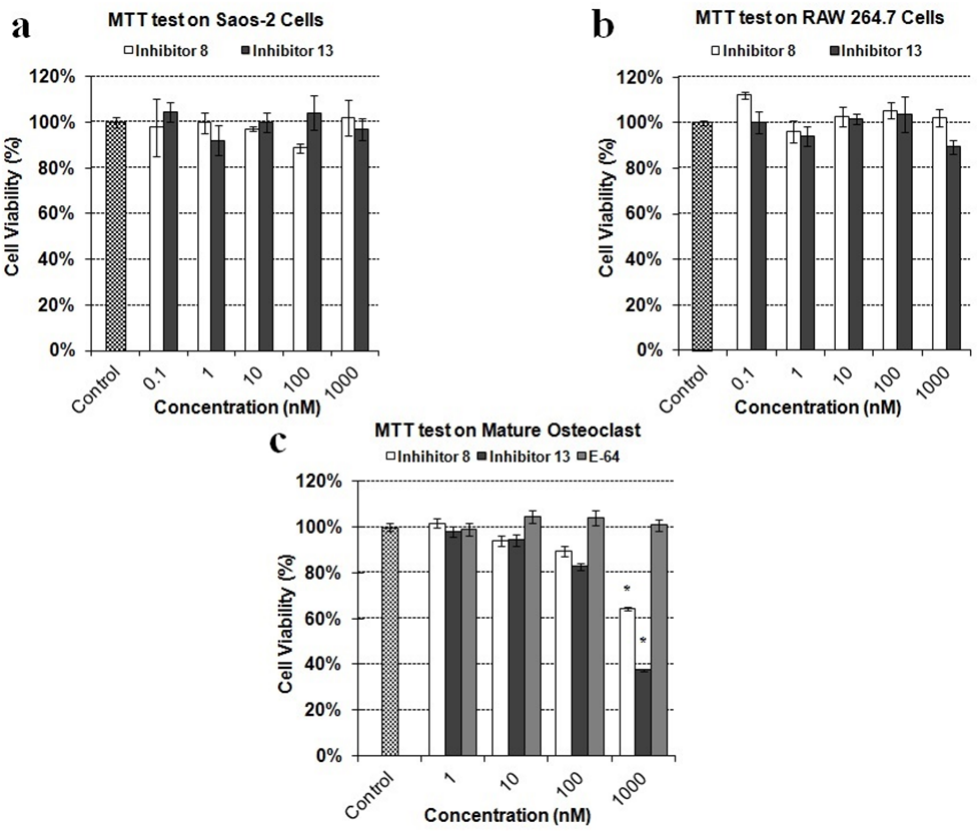
Cytotoxicity of CatK inhibitors. MTT test of **CKI-8** and **CKI-13** on a) osteoblast-like Saos-2, b) RAW 264.7 and c) mature osteoclasts, respectively, at the indicated concentrations. Results are expressed as percentage of control containing DMSO 0.1% (mL: mL) without inhibitor. Three independent measurements were repeated in triplicate for each inhibitor (n = 9). A t test was performed to evaluate significant differences. * *p* value <0.05 was regarded as statistically significant (comparison with the control).

The cytotoxicity of CKI were tested on three distinct types of cells: human osteoblast-like Saos-2, mouse monocyte cell line RAW 264.7 and mature mouse osteoclast cells, using an MTT assay. These tests were performed after the cells had been incubated without (control) or with the compounds for 3 days. CKI-8 and CKI-13 were not toxic in the concentration range from 0.1 to 1000 nM on osteoblast-like Saos-2 cells (Fig 2A) and on RAW 264.7 cells (Fig 2B). On mature mouse osteoclasts differentiated from bone marrow, there was no toxicity up to 100nM, while at 1000 nM, less than 40–60% cells were viable (Fig 2C). On the other hand, the commercial inhibitor E64 was not toxic throughout this concentration range (Fig 2C). The addition of 0.1% (mL: mL) DMSO used to solubilize the inhibitors did not affect cell toxicity.

### Mineralization of osteoblast-like Saos-2 cells and primary mouse calvarial osteoblasts

**Fig 3:**
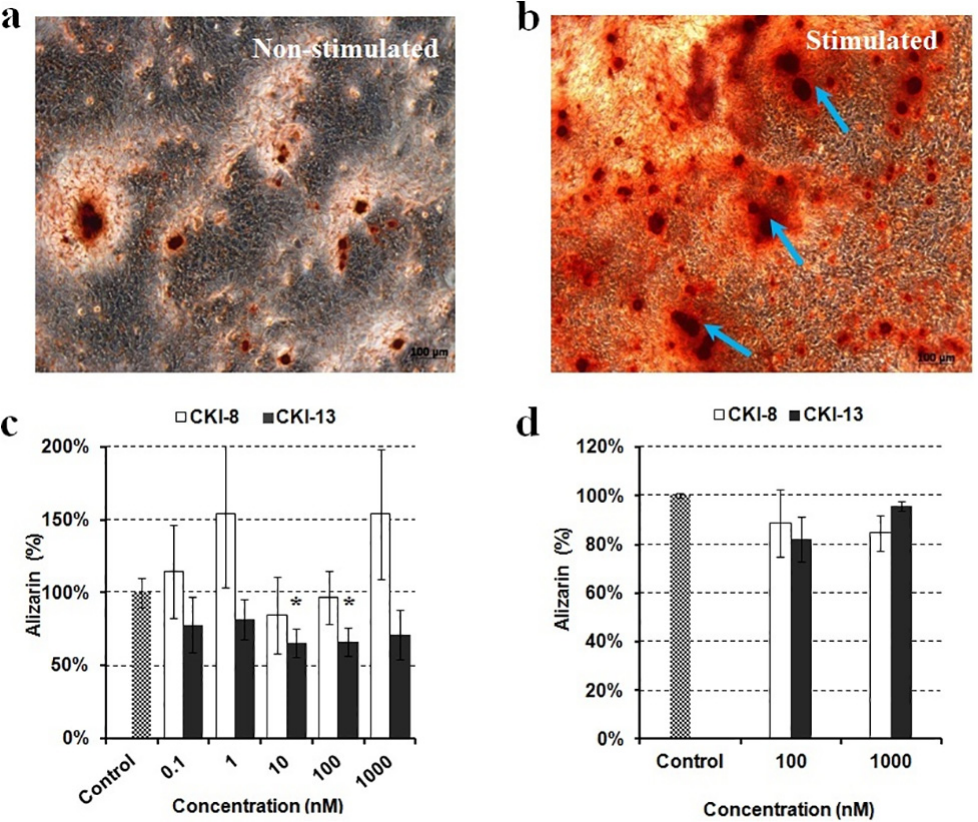
Effect of the two catK inhibitors on AR-S staining, induced by Saos-2 cells and osteoblasts from calvaria. Typical example of AR-S staining on Saos-2 cells incubated during eight days a) without or b) with the addition of 50 **μ**g mL^-1^ AA and 7.5 mM β-GP. The effects of control (without inhibitor in grey rectangles), **CKI-8** (unfilled rectangles) and **CKI-13** (black rectangles) on mineralization as indicated by their concentrations were monitored by AR-S staining on c) Saos-2 cells and d) primary osteoblasts. A t test was performed to evaluate significant differences. * *p* value <0.05 was regarded as statistically significant (comparison with the control).

**Fig 4:**
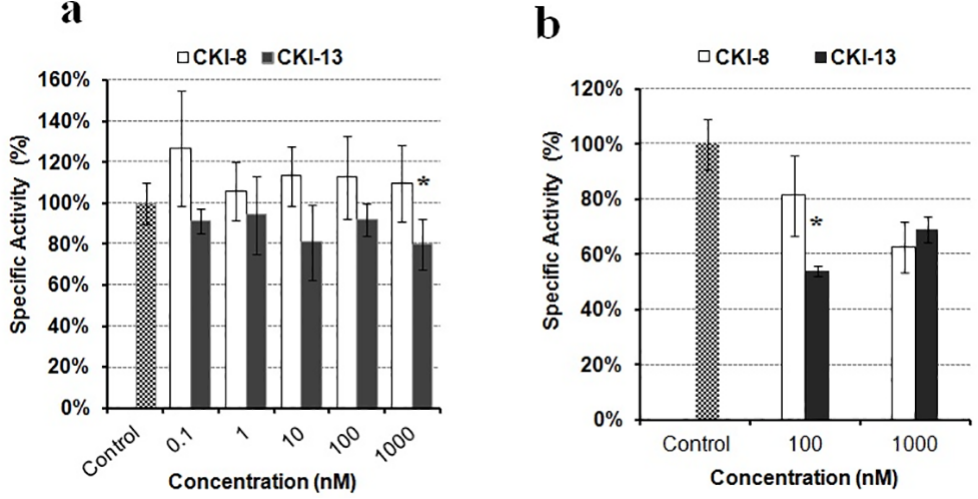
Effect of the two CatK inhibitors on TNAP activity in Saos-2 cells and osteoblasts from calvaria. Their effects on TNAP activity as functions of the indicated concentrations were measured in a) Saos-2 cells and in b) primary cells. Results are expressed as percentage of control containing DMSO 0.1% (mL:mL) without inhibitor. Three independent measurements were repeated in triplicate for each inhibitor (n = 9). A t test was performed to evaluate significant differences. * *p* value <0.05 was regarded as statistically significant, (comparison with the control).

To check whether **CKI-8** or **CKI-13** could inhibit the mineralization process, Saos-2 cells and osteoblasts isolated from mouse calvaria were selected as cell models to monitor mineralization. To stimulate mineralization, 50 **μ**g mL^-1^ AA and 7.5 mM β-GP, as osteogenic factors, were added [27–29]. The extent of AR-S staining was measured to quantify calcium nodules (Fig 3A and 3B). As reported earlier [30], mineralization induced by Saos-2 cells was strongly enhanced by the combined addition of AA and *β*-GP in the cell cultures (Fig 3B) as compared without any addition of osteogenic factors (Fig 3A). We determined the effects of the inhibitors **CKI-8** and **CK-13** on the mineralization process induced by Saos-2 cells (Fig 3C) and by osteoblasts, (Fig 3D). As shown by AR-S staining, **CKI-13** significantly inhibited mineralization with Saos-2 cells at 10 and 100 nM concentrations to around 60% of control (Fig 3C, black rectangle), while **CKI-8** did not inhibit significantly, although there were slight variations (Fig 3C, unfilled rectangles). We then measured the effect of **CKI-8** and **CKI-13** on the activity of TNAP, a mineralization marker [28], in cells. **CKI-13** reduced TNAP activity significantly on Saos-2 cells at 1000 nM to 80% of control (Fig 4A, black rectangles), suggesting that **CKI-13** can slow down TNAP activity and mineralization. **CKI-8** did not decrease TNAP activity over the concentration range from 0.1 to 1000 nM (Fig 4A, unfilled rectangles). We determined the effects of both **CKI-8** and **CKI-13** at concentrations ranging from 100 to 1000 nM in osteoblasts from mouse calvaria. AR-S staining did not indicate any significant effect of either **CKI-8** or **CKI-13** on cell-induced mineralization in osteoblasts (Fig 3D). However, TNAP activity decreased from 100% (control) to 58% of control using 100 nM **CKI-13** (Fig 4B). In contrast, **CKI-8** did not inhibit TNAP significantly, consistent with the AR-S staining results. In all cases, the addition of 0.1% (mL:mL) DMSO to solubilize the inhibitors did not affect calcium nodule formation and TNAP activity.

### Gelatin zymography assay on cathepsin K activity in osteoclast differentiated from RAW 264.7 or from bone marrow

**Fig 5:**
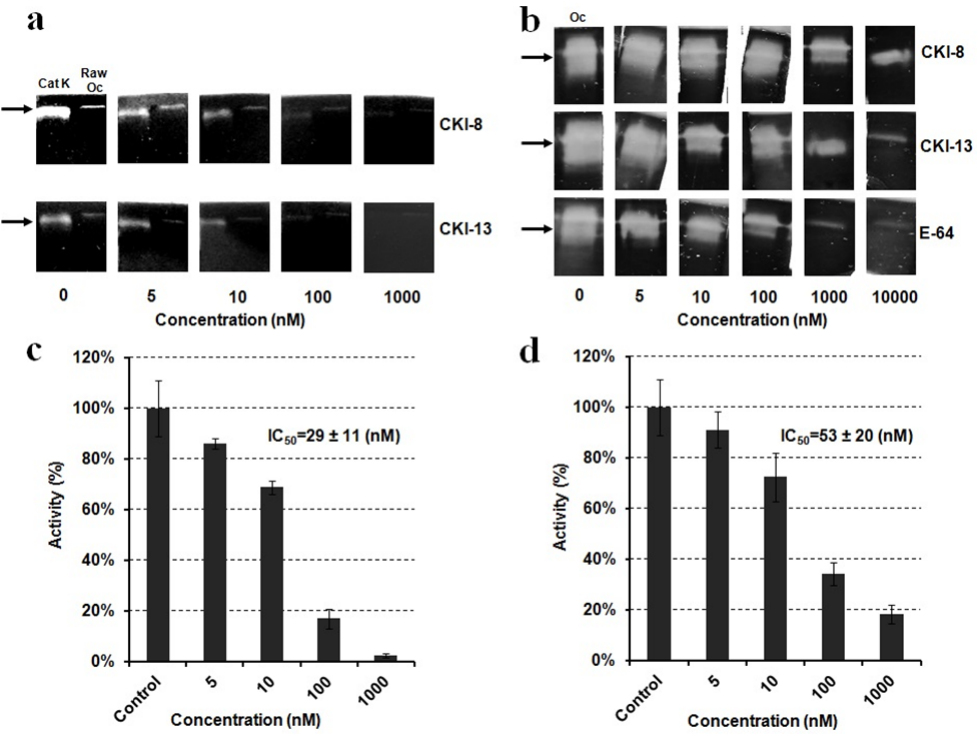
Gelatin zymography assay on CatK activity. a) Left lanes: Human recombinant CatK as positive control and right lanes: CatK extracted from RAW264.7 cells (RAW OC) treated with 0,5,10,100,1000 nM of CatK inhibitors (**CKI-8** and **CKI-13**); b) CatK (OC) extracted from osteoclasts differentiated from bone marrow and treated with 0,5,10,100,1000 nM of CatK inhibitors (**CKI-8** and **CKI-13**); c) IC_50_ value of **CKI-13** and IC_50_ value **CKI-8** (d) as determined by zymography (twice repeated) using human CatK.

We wanted to verify whether CKIs could inhibit mouse CatK from extracellular medium of osteoclast-like cells differentiated from RAW 264.7 or from osteoclasts differentiated from bone marrow cells. Recombinant human CatK was used as a control (600 ng) and as a protein marker in zymography. Cellular proteins (300 ng) including CatK were extracted from extracellular media of osteoclasts differentiated from RAW 264.7 or from bone marrow cells. Human CatK (Fig 5A, left lanes) migrated at the same position as CatK in RAW 264.7 cell extracts on the zymography gel (Fig 5A, right lanes). Gels were then pre-incubated with **CKI-8** (Fig 5A, top lanes) and **CKI-13** (Fig 5A, bottom lanes), respectively, at concentration ranging from 5 to 1000 nM. At 1000 nM, both inhibitors were able to completely block the gelatinolytic activity of human CatK (Fig 5A, left lanes) or mouse CatK (Fig 5A, right lanes). We also observed a slight inhibition of gelatinolytic activity of CatK in extracts of osteoclasts differentiated from bone marrow cells (Fig 5B), albeit less pronounced than observed for RAW 264.7 cells or human CatK (Fig 5A). From the results of gelatin zymography, it is possible to calculate the apparent IC_50_ of inhibition of **CKI-8** and **CKI-13** on human CatK (Fig 5C and 5D). A value of IC_50_ = 51 ± 20 nM was calculated for **CKI-8**, and a value of IC_50_ = 29 ± 11 nM was obtained for **CKI-13**. The addition of 0.1% (mL:mL) DMSO used to solubilize the inhibitors did not affect the inhibition.

### Assessment of bone resorption index on bone model matrix

**Fig 6:**
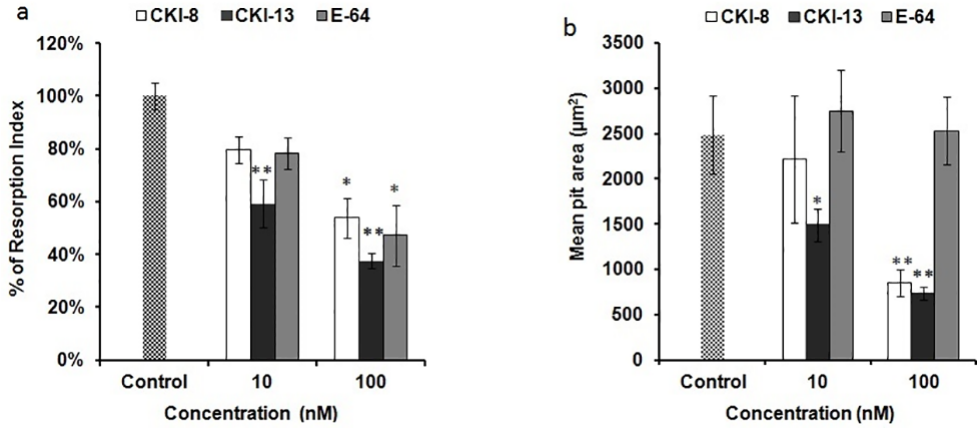
Resorption activity of osteoclasts on bovine bone slices with or without cathepsin K inhibitors. a) Resorption index of osteoclasts differentiated from bone marrow with 0.1% DMSO (mL:mL) not treated (control) or treated with following inhibitors: **E-64**, **CKI-13** and **CKI-13**. b) Quantitative analysis of mean pit area (μm^2^) per number of osteoclast on bovine bone slices without and with inhibitors at 100 nM. A t test was performed to evaluate significant differences. Three distinct sample pools were analyzed in a duplicate manner (n = 6). * *p* value <0.05 and ** *p* <0.01 were regarded as statistically significant (comparison with the control).

**Fig 7:**
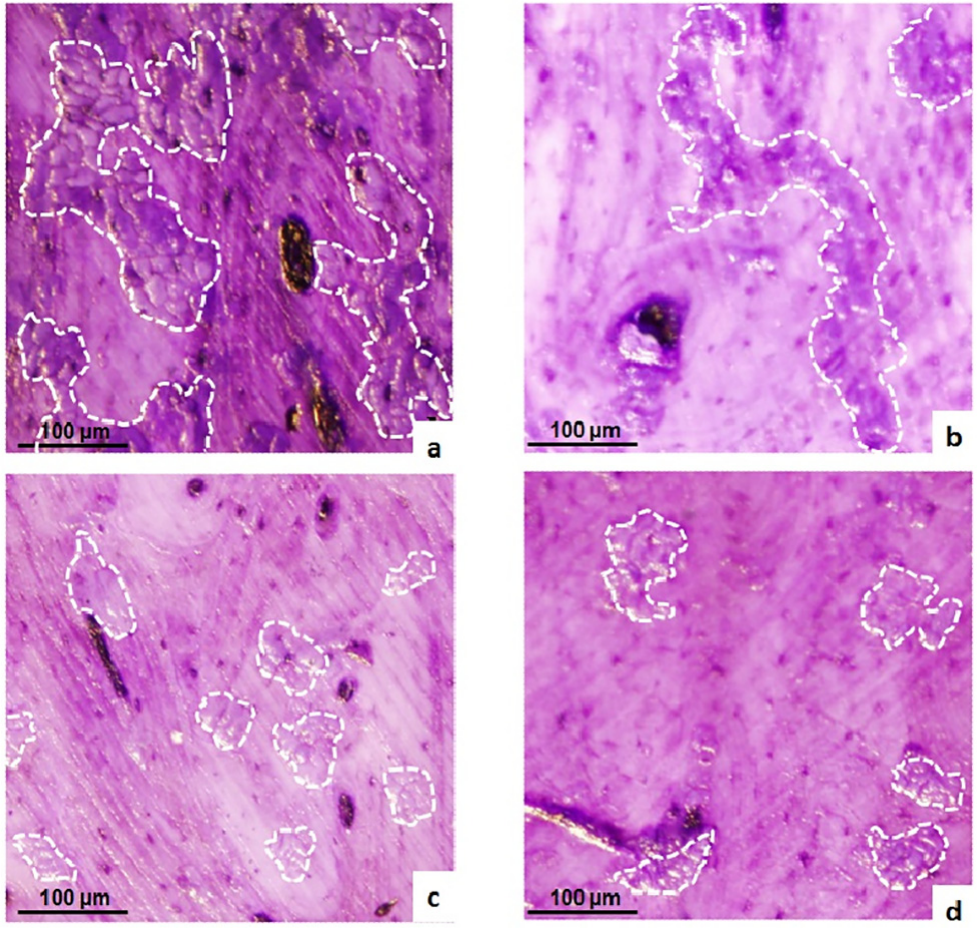
Resorption pits induced by mature osteoclasts with or without cathepsin K inhibitors. Pits as indicated by white contours were formed by osteoclasts resorbing on bovine bone plates without inhibitors (a), with **E-64** (b), with **CKI-8** (c) and with **CKI-13** (d) at 100 nM. A t test was performed to evaluate significant differences. Three distinct sample pools were analyzed in a duplicate manner (n = 6). * *p* value <0.05 and ** *p* <0.01 were regarded as statistically significant (comparison with the control).

Since CatK is a collagenase and the predominant papain-like cysteine protease expressed in osteoclasts, [5, 6] and that type I collagen constitutes the major part of bone organic matrix, we evaluated the effects of CKI-8 and CKI-13 on mature osteoclast resorbing bovine bone slices. Mononuclear cells from bone marrow were seeded in complete α-MEM medium supplemented with 30 ng mL^-1^ mRANKL and 25 ng mL^-1^ recombinant mouse macrophage-colony stimulating factor for five days. Then the mature osteoclasts were treated with or without CKIs. The results showed that CKI-13 at 10 and 100 nM, while CKI-8 and E64 both at 100nM decreased resorption significantly by osteoclasts on bovine bone slices (Fig 6A). Significant difference in the mean area of resorption pits was observed between CKI-8, CKI-13 and E64 at 100nM (Fig 6B). Moreover, the resorption lacunae on bovine bone slices without any inhibitor were clearly larger (Fig 7A) than the resorption lacunae on bovine bone slices treated with E64 (Fig 7B), or with CKI-8 (Fig 7C) or with CKI-13 (Fig 7D).

## Discussion

We reported the properties of a series of cathepsin K, B, L and S inhibitors, among these CKI-8 and CKI-13 being highly selective for CatK over B and S [24]. As these inhibitors were selective under *in vitro* conditions using human recombinant CatK as a target enzyme, zymography was used to ascertain their inhibition properties on mouse CatK. Both CKI-8 and CKI-13 inhibited CatK in extracts of osteoclasts differentiated from RAW 264.7 or from bone marrow at higher concentrations than expected from *in vitro* values [24]. The IC_50_ values determined from zymography of CKI-8 (51 ± 20 nM) and of CKI-13 (29 ± 11 nM) were higher than those of CKI-8 (0.263 nM) and CKI-13 (0.006 nM) acting on human CatK determined using the colorimetric method on dipeptides containing fluorophore [24]. This discrepancy is due to several factors. 1) The substrate dipeptides (used for UV-visible spectroscopy) and gelatin (used for zymography) are different in size and structure, affecting their accessibility to the active site of CatK, as well as the inhibitor’s binding site. 2) Human CatK recombinant enzyme and mouse CatK extracted from cells may interact differently with CKI inhibitors. 3) Alternatively gel zymography might overestimate the apparent IC_50_ due to the long exposure of inhibitors. 4) Since IC_50_ depends of the substrate concentration and that concentration of gelatin is relatively high as compared to that of the inhibitor, the apparent IC_50_ can also be overestimated. To validate the feasibility of the possible therapeutic use of the candidate inhibitors prior to preclinical trials, we tested their toxicities, their effects on the mineralization process induced by osteoblasts and their influence on resorption mediated by osteoclasts. CKI-8 and CKI-13 showed no toxicity up to 1000 nM on osteoblast-like Saos-2 cells and on RAW 264.7 cells. However, they were toxic when their concentrations exceeded 100 nM in osteoclasts differentiated from bone marrow. This suggested that the maximum concentration in cells without producing any cytoxicity was around 100 nM for the candidate molecules. Neither CKI-8 nor CKI-13 affected the mineralization process induced by primary osteoblasts although a slight inhibition of TNAP activity by CKI-13 was observed. CKI-13 slowed down significantly the mineralization process induced by the Saos-2 cell line, as well as inhibiting slightly the TNAP activity. The different effects on mineralization of CKI-13 on both types of cells are probably due to their distinct maturation times, as well as their mouse vs human origin. Bone resorption on bovine slices decreased significantly at 10 nM CKI-13, while 100 nM CKI-8 and 100 nM of the commercial inhibitor E64 were necessary to significantly decrease bone resorption. CKI-8 and CKI-13 treatments produced smaller pits (Fig 7C and 7D) when compared with the larger pits induced by E64 treatment (Fig 7B), suggesting that CKI-8 and CKI-13 might affect the mobility of osteoclasts in addition to their osteoclast resorption activity. Possible targets of CKI-8 and CKI-13 are F actin and other proteins involved in osteoclast podosome dynamics [38]. Although the findings look promising, especially the bone-resorption property of CKI-13, further work is needed to ascertain the feasibility of using CatK inhibitors to treat bone resorption diseases, since their side effects on TNAP activity may impair their therapeutic potential, especially for CKI-13. On the other hand, the side effect of CKI-13 by producing a reducing mineralization might slow down bone resorption due to a coupling signaling between osteoblasts and osteoclasts. This has been observed on the reverse direction [39–41] when bisphosphonates and the anti RANK antibody denosumab produce a marked reduction of bone formation, following the inhibition of bone resorption [42]. Co-cultures of osteoblasts and osteoclasts, as well as animal-model trials need to be performed to strengthen the feasibility of using these small molecules to prevent bone resorption.
